# *XDSGUI*: a graphical user interface for *XDS*, *SHELX* and *ARCIMBOLDO*

**DOI:** 10.1107/S1600576723007057

**Published:** 2023-09-05

**Authors:** Wolfgang Brehm, Josep Triviño, Juno M. Krahn, Isabel Usón, Kay Diederichs

**Affiliations:** a Department of Physics, University of Hamburg, Hamburg 22761, Germany; b Instituto de Biologia Molecular de Barcelona (IBMB), Baldiri Reixach 15, Barcelona 08028, Spain; c Genome Integrity and Structural Biology Laboratory, National Institute of Environmental Health Sciences, Research Triangle Park, NC 27709, USA; d ICREA: Institució Catalana de Recerca i Estudis Avançats, Pg. Lluis Companys 23, Barcelona 08010, Spain; e Department of Biology, University of Konstanz, Universitätsstrasse, Konstanz 78457, Germany; DESY, Hamburg, Germany

**Keywords:** X-ray diffraction, neutron diffraction, electron diffraction, data processing, graphical user interfaces, phasing, *XDS*, *ARCIMBOLDO*, *SHELX*

## Abstract

*XDSGUI* is a lightweight graphical user interface (GUI) for the *XDS*, *SHELX* and *ARCIMBOLDO* program packages that serves both novice and experienced users in obtaining optimal processing and phasing results for X-ray, neutron and electron diffraction data. The design of the program enables data processing and phasing without command line usage, and supports advanced command flows in a simple user-modifiable and user-extensible way. The GUI supplies graphical information based on the tabular log output of the programs, which is more intuitive, comprehensible and efficient than text output can be.

## Abstract 2

A customizable stateless graphical user interface simplifies the processing, analysis and phasing of diffraction data.

## 1. Introduction

The development of the *XDS* data processing package (Kabsch, 2010*a* ▸,*b* ▸) and the *SHELX* (Sheldrick, 2008 ▸) phasing programs was started about 40 and 50 years ago, respectively, at a time when computers were widely available in laboratories working in crystallography but graphics terminals were expensive and scarce. For this reason, the programs were executed through commands entered in a text-only terminal and by editing of input files. Their output was and still is human-readable ASCII files, with additional information, warnings and errors displayed on the terminal.

*ARCIMBOLDO*, a program package for fragment-based phasing through molecular replacement and expansion, was developed much later (Rodríguez *et al.*, 2009 ▸; Millán *et al.*, 2015 ▸). It builds upon the programs *SHELXE* and *Phaser* (McCoy *et al.*, 2007 ▸; Oeffner *et al.*, 2018 ▸) and its development was started at a time when a cluster of computers was needed for running parallel computations.

The benefits of a graphical user interface (GUI) are evident, and they may be combined with the control and flexibility of the command line by integrating its functionality within the interface to mediate efficient use of these computational program packages. Here we describe *XDSGUI*, a Qt-based graphical interface which allows users to analyze data sets and solve structures by running the programs without having to use the command line in a terminal window. Since *XDSGUI* provides suitable defaults wherever possible but makes all features available, setting up the computational programs requires only minimal user input, while maximizing control and customizability. The computational programs can be started by a mouse click and their output is automatically displayed in graphical form. *XDSGUI* thus eases and speeds up the assessment of data and models, and the detection of errors and problems during structure solution.

For *XDS* data processing, a GUI is implemented in *e.g.* *XDSAPP* (Sparta *et al.*, 2016 ▸; current version at https://www.helmholtz-berlin.de/xdsapp) with an emphasis on automation. For *SHELX* phasing, the first graphical interface, still available, was *HKL2MAP* (Pape & Schneider, 2004 ▸). A sophisticated and thorough exploration of parameter space is available through *SHELIXIR* (Kolenko *et al.*, 2021 ▸). The *CCP4* (Winn *et al.*, 2011 ▸; Agirre *et al.*, 2023 ▸) interfaces *CCP4i* (Potterton *et al.*, 2003 ▸), *CCP4i2* (Potterton *et al.*, 2018 ▸), *CCP4Cloud* (Krissinel *et al.*, 2022 ▸) and *CCP4 Online* (Krissinel *et al.*, 2018 ▸) integrate *SHELX* and *ARCIMBOLDO*, also maximizing automation of operation.

Automation is particularly useful for good data sets, whereas the interactivity of *XDSGUI* is an advantage for poor or difficult data sets, since alternative processing and phasing regimes can be easily explored.

The purpose of this paper is to explain the basic ideas behind *XDSGUI*. Detailed documentation can be found online in the XDSwiki (https://wiki.uni-konstanz.de/xds/index.php/XDSGUI). Although *XDSGUI* facilitates usage of the computational programs, prior knowledge of them is advantageous for the successful use of *XDSGUI*. Worked-out data processing examples are available at https://wiki.uni-konstanz.de/xds/index.php/Quality_Control.

## 2. Materials and methods

### 2.1. Implementation, availability and installation

*XDSGUI* is written in C++ using the Qt libraries (https://www.qt.io/) and *QCustomPlot* (https://www.qcustomplot.com/). The scripts that add flexibility to *XDSGUI* use the shell, which is typically bash on Linux and zsh on macOS. The first version of the program, which only supported *XDS* data processing, was released in 2013 (https://www.jiscmail.ac.uk/cgi-bin/wa-jisc.exe?A2=CCP4BB;124022c8.1306); *SHELX* and *ARCIMBOLDO* were integrated in 2018 and 2019, respectively. Program development is ongoing and we appreciate user feedback.

The source code of *XDSGUI* is licensed under GNU General Public License version 3.0 (GPLv3) and available from https://sourceforge.net/u/joseptrivino/xdsgui/ci/master/tree/. Besides a C++ compiler, the Qt headers and libraries must be available for compiling and linking. For non-commercial use, binaries for Linux/x86_64, macOS/x86_64 and macOS/arm64 are provided from the XDSwiki web page https://wiki.uni-konstanz.de/xdswiki/index.php/XDSGUI. Installation on Linux means copying the binary into a directory that is in the $PATH of the user, and may require installing Qt dependency libraries on the user’s system. On macOS, the program should be installed under /Applications, as is usual on this operating system, and it should be linked to /usr/local/bin/xdsgui as suggested in the *Installation* article of the XDSwiki (https://wiki.uni-konstanz.de/xds/index.php/Installation).

Data processing with *XDSGUI* primarily requires the *XDS* program package (https://xds.mr.mpg.de/). The script *generate_XDS.INP* is available from https://wiki.uni-konstanz.de/xds/index.php/Generate_XDS.INP for preparing XDS.INP, the input file for the *XDS* main program. Additional features of *XDSGUI* make use of the programs *XDSCC12* (Assmann *et al.*, 2016 ▸), *XDSSTAT* (Diederichs, 2006 ▸), *Coot* (Emsley *et al.*, 2010 ▸), *POINTLESS* (Evans, 2011 ▸) and auxiliary programs from the *CCP4* suite; these are available from or linked to the *Installation* article of the XDSwiki. Comparison of current and previous versions of CORRECT.LP in the ‘tools’ tab requires a program to visualize differences between text files; the name of that program (*e.g. xdiff*, *xxdiff*, *tkdiff* or *vimdiff*) may be adjusted according to local availability in the script box below the button. Phasing with *XDSGUI* requires the *SHELX* and *ARCIMBOLDO* programs, which are available standalone or as part of the *CCP4* program suite. *ARCIMBOLDO* requires *Phaser*, available also through *CCP4*.

Configuration of display fonts, plot sizes, program paths and libraries required for HDF5 data processing takes place in Menu/Settings on Linux or Menu/Preferences on macOS.

An apparent limitation of *XDSGUI* is that the names of the data files, including the path to them, should not embed blank characters, *e.g.* as in ‘My USB disk’. This results from the filename treatment of the computational programs and is not expected to be changed anytime soon. If the file or directory names cannot be changed, the workaround is to use Unix symbolic links.

### 2.2. Functional description

*XDSGUI* should ideally be started from a terminal window to see the output of helper programs (like *POINTLESS* or *Coot*), and possibly error messages from *generate_XDS.INP*, *XDSSTAT* or *XDSCC12*. The program may be used for graphical analysis of any existing files and subdirectories written by *XDS*, *SHELX* and *ARCIMBOLDO*, or may be used to create these files.

The main function of *XDSGUI* is to mediate between command-line-driven computational programs, reading and writing ASCII output files, and a user who expects easy configuration of the computational programs and meaningful information about processing results. To achieve this, all data exchange between *XDSGUI* and the computational programs is through files, as documented for the respective programs. This means that *XDSGUI* is stateless: the information it displays depends on the data on disk rather than on the sequence of operations performed. Another benefit is that a crash of *XDSGUI* does not compromise the computational programs, and *vice versa*.

User interaction, on the other hand, occurs through *XDSGUI* buttons labeled *e.g.* ‘Run XDS’ or ‘Run SHELXC’ or, in the case of more complex operations, labeled buttons that run a script. These scripts are shown as one line of editable text below the respective buttons, and consist of shell commands that are separated from each other by semicolons. The scripts are usually short, but in principle they may be arbitrarily long, and can invoke programs or other scripts. This allows a productivity enhancement through leveraging of external programs that are not part of the computational program packages – these programs become available through the one-line scripts. Default scripts are provided, and they can be simply edited by changing or deleting existing commands and/or inserting new commands.

Editing the scripts constitutes a simple way of customizing and extending *XDSGUI*. Some buttons and their associated one-line scripts are currently unused and could *e.g.* be used to run additional software. For example, the empty script associated with a default button labeled ‘User-defined command’ could be changed to ‘phenix.xtriage XDS_ASCII.HKL’ in order to run a *Phenix* (Liebschner *et al.*, 2019 ▸) command, and the label of the button could be changed to ‘Analyze data with Phenix’. Even better would be ‘xterm -e ’phenix.xtriage XDS_ASCII.HKL; echo wrote xtriage.log; cat’; mv -f logf ile.log xtriage.log’, since that would direct the output to a new terminal window and save it in xtriage.log.

Default (and potentially edited) shell scripts, together with their button labels, are automatically stored in $HOME/.xds-gui, which is the only file that is particular to *XDSGUI*.

### 2.3. Overview of graphical elements in *XDSGUI*

The data representations and operations enabled by *XDSGUI* are shown in individual tabs arranged horizontally along the upper edge of the *XDSGUI* window. *XDSGUI* has the concept of a ‘project folder’, which is the directory that receives the files of the current data processing and phasing run; it should be separate from the directory where the raw data are stored. The project folder is shown in the title bar of the *XDSGUI* window and can be changed in the first tab (labeled ‘Projects’). The project folder contains subdirectories for *SHELX* and *ARCIMBOLDO*, respectively.

**Figure 1:**
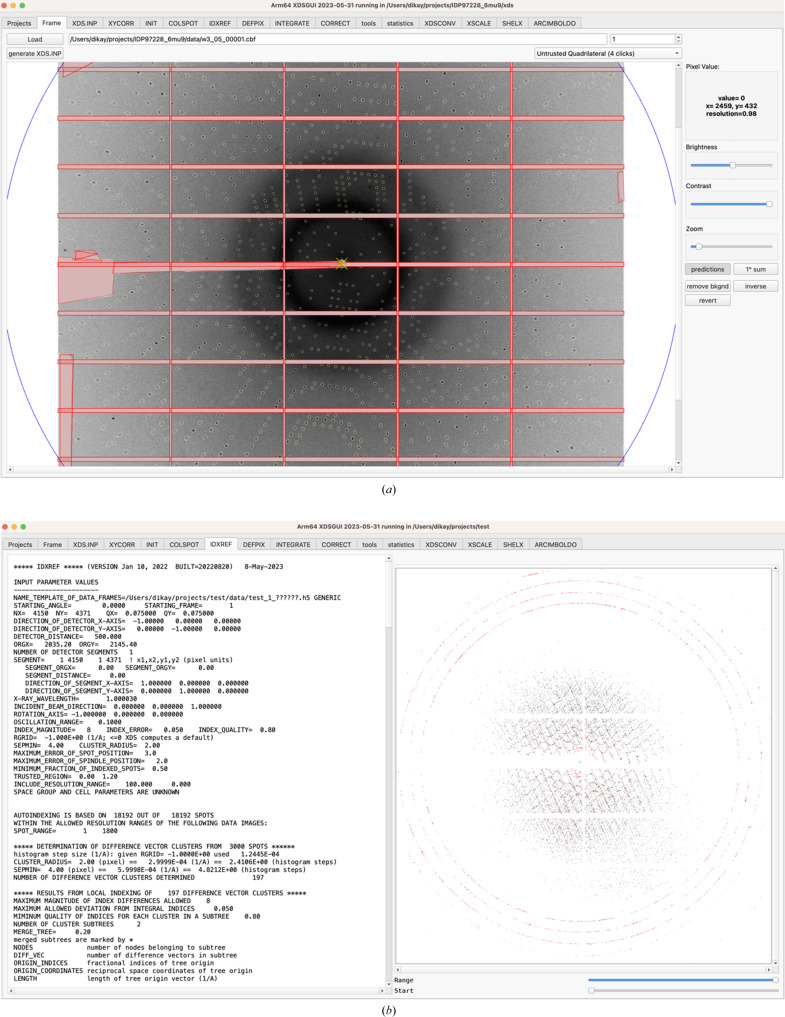
Input and output of *XDS*. (*a*) The ‘Frame’ tab. Shadowed detector areas are masked (red semi-transparent areas) and circles indicate predicted reflections. (*b*) The ‘IDXREF’ tab showing, as a projection on the detector, the non-indexed reflections in red and the indexed reflections in black. Ice rings, not visible in the raw data frames, are clearly seen.

The second tab, labeled ‘Frame’ [Fig. 1 ▸(*a*)], serves (i) to load and visualize a data image from a data directory, with the possibility of showing predicted spot positions once IDXREF has succeeded, (ii) to generate, on the basis of the header of the loaded image, an XDS.INP file suitable for the detector used, and (iii) to mask untrusted areas of the detector. In addition, we recently implemented the optional addition of adjacent fine-sliced frames to ‘Virtual 1-degree frames’ for visualization, so that users can be reassured that they do not miss any pathology that only becomes visible at stronger per-frame exposure.

**Figure 2:**
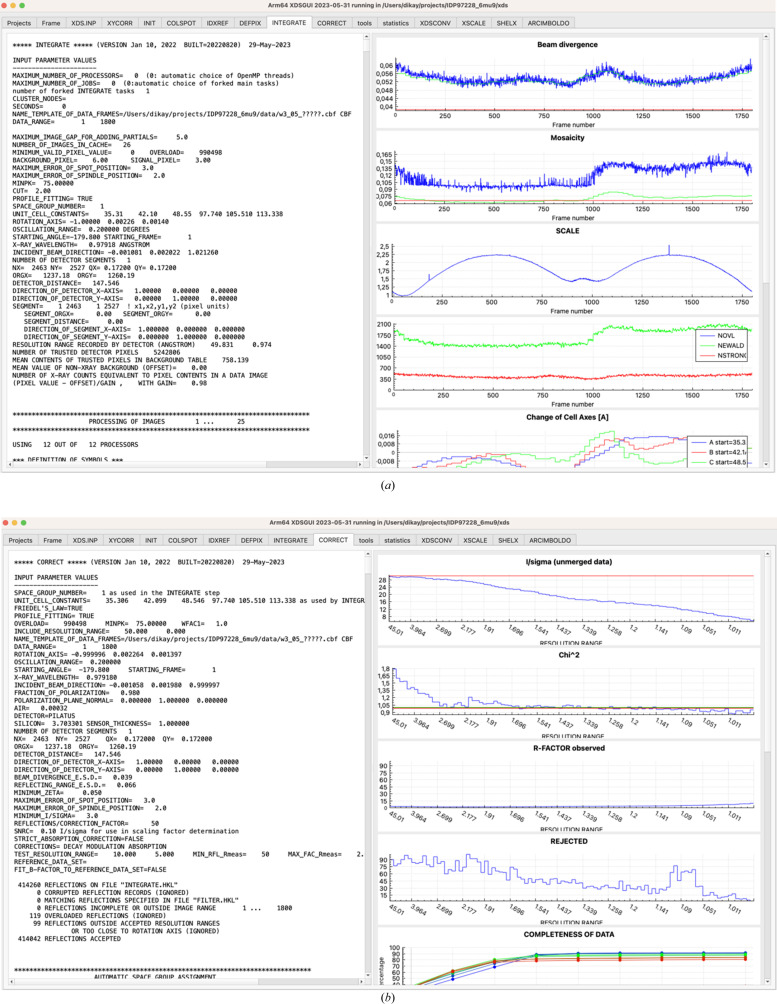
Input and output of *XDS*. (*a*) The ‘INTEGRATE’ tab. Only the upper five out of eight plots are shown. Some instrumental instability is detected as a discontinuity near frames 180 and 1380 of the ‘SCALE’ plot. (*b*) The ‘CORRECT’ tab. Only the upper five out of eight plots are shown. ISa is represented by the red line in the uppermost plot; a value near 30 in this example indicates a low level of systematic error. The nine different colors, from blue to red, in the ‘completeness’ and following plots show the variation of the indicator from low resolution to high resolution.

**Figure 3:**
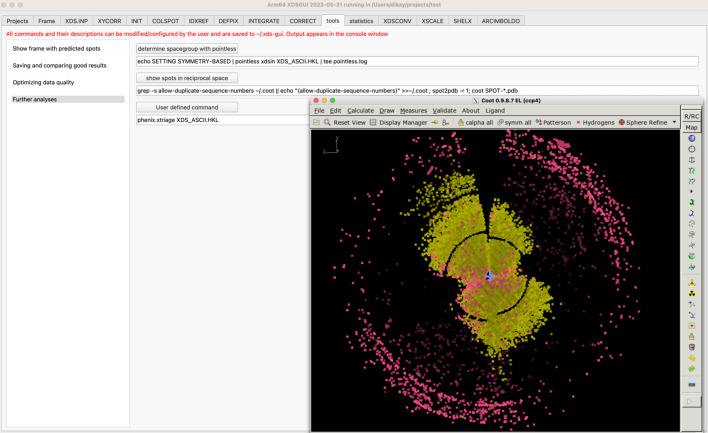
The ‘tools’ tab showing the ‘Further analyses’ option. The button ‘show spots in reciprocal space’ executes a shell script (shown below the button). As a result, *Coot* provides a reciprocal-space view of indexed (yellow) and non-indexed (pink) reflections. Ice rings and the borders between detector modules are prominent. These are the same data as displayed in Fig. 1 ▸(*b*).

**Figure 4:**
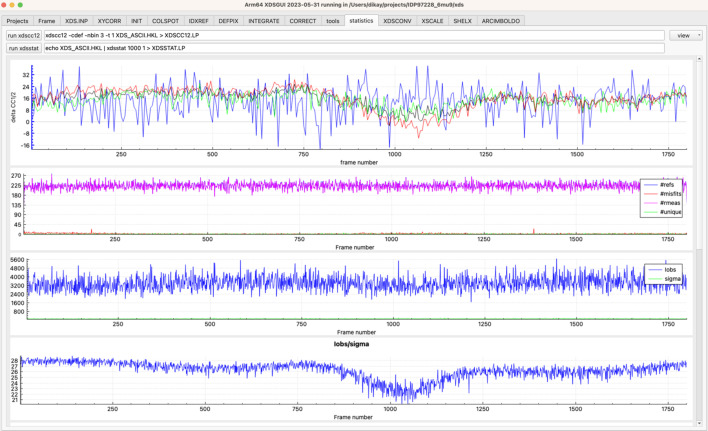
The ‘statistics’ tab. Four out of eight plots are shown. (Top) Δ-CC_1/2_ plot obtained by removing 1° of data (the -t 1 option of *XDSCC12*) and observing the difference in CC_1/2_ with and without these data on the overall CC_1/2_ in a resolution range (blue = low resolution, red = high resolution, green = intermediate resolution, black = overall). The high-resolution data near frame 1070 have a negative Δ-CC_1/2_, indicating that they reduce the quality of the overall CC_1/2_ at high resolution. The user may remove these data from processing by including the line EXCLUDE_DATA_RANGE = 1000 1100 in XDS.INP, but a compromise with respect to completeness must be found. Further plots are from *XDSSTAT*; the bottom plot shows that the overall *I*/σ drops from about 28 to 21 in this frame range.

The next tab is labeled ‘XDS.INP’ and features a simple editor. It also lets the user start *XDS* and inspect its terminal output. The following tabs (‘XYCORR’ for geometric corrections, ‘INIT’ for intial background estimation, ‘COLSPOT’ for spot finding, ‘IDXREF’ for indexing, ‘DEFPIX’ for masking, ‘INTEGRATE’ for integration of reflections, and ‘CORRECT’ for spacegroup determination and scaling) are named according to the processing steps of *XDS* (Kabsch, 2010*a* ▸) and display its output files. The ‘COLSPOT’, ‘IDXREF’ [Fig. 1 ▸(*b*)], ‘INTEGRATE’ [Fig. 2 ▸(*a*)] and ‘CORRECT’ [Fig. 2 ▸(*b*)] tabs additionally present plots of tabular data. The ensuing ‘tools’ (Fig. 3 ▸) and ‘statistics’ (Fig. 4 ▸) tabs give access to other crystallographic programs, visualize their output and allow optimization of the data.

Rightmost, the tabs ‘XDSCONV’, ‘XSCALE’, ‘SHELX’ and ‘ARCIMBOLDO’ allow access to the named computational programs and their input and output.

The user is generally advised to inspect and use the tabs from left to right, *i.e.* in the order of their contents being produced or available. Some tabs are grayed out if the corresponding data files are not (yet) available. Tabs holding currently running computations are highlighted in green.

Plots can be resized by clicking and dragging their border with the mouse, and can be saved as a PDF or PNG image using a right-click. Clicking on an axis or into the plot and then scrolling with the middle mouse wheel allows enlarging of that plot dimension or the entire plot, and click and drag of the plot moves it on the canvas.

## 3. Results and discussion

### 3.1. Usage for *XDS* data processing

For processing of raw data, the user starts with the choice of the processing directory (‘Projects’ tab) and then advances to the ‘Frame’ tab to display a raw data frame that is loaded from a data directory. Within the ‘Frame’ tab, XDS.INP may be generated; for this to work, a raw data frame must be displayed first.

While still in the ‘Frame’ tab [Fig. 1 ▸(*a*)], untrusted (shaded) areas may be marked with the mouse, which will write the appropriate lines to XDS.INP; ORGX ORGY may be corrected if necessary, and the detector areas corresponding to INCLUDE_RESOLUTION_RANGE, EXCLUDE_RESOLUTION_RANGE and TRUSTED_REGION may be inspected or adjusted.

The latter operations are interactive in the sense that the user modifies the values in the ‘XDS.INP’ tab and then checks the result in the ‘Frame’ tab, and a change in either is mirrored in the other. In other words, the user moves back and forth between the ‘Frame’ and the ‘XDS.INP’ tabs.

From the ‘XDS.INP’ tab, other parameters, *e.g.* SPACE_GROUP_NUMBER and UNIT_CELL_CONSTANTS, can be adjusted by the user, and finally *XDS* may be run by clicking a button. The *XDS* log file is then available from the ‘XDS.INP’ tab and its output files are displayed in the next tabs.

The first round of processing produces data and their visualization in the ‘COLSPOT’, ‘IDXREF’ [Fig. 1 ▸(*b*)], ‘DEFPIX’, ‘INTEGRATE’ [Fig. 2 ▸(*a*)] and ‘CORRECT’ [Fig. 2 ▸(*b*)] tabs. Subsequently, the ‘tools’ tab allows the user to analyze the processed data with other programs, most notably *POINTLESS* for space-group analysis and *Coot* for visualization of indexed and non-indexed reflections in reciprocal space (Fig. 3 ▸), which often reveals reasons for failure of indexing.

The ‘tools’ tab also offers three options that have been found useful to optimize the data processing results. After choosing (by mouse click) one of these three options, the user should go back to the ‘XDS.INP’ tab, specify JOBS = DEFPIX INTEGRATE CORRECT and run *XDS* again.

Each of the three options should be tried separately and its effect should be assessed, using a tool that visualizes differences between text files, to verify that it really improved the processed data. The values of the indicator for the systematic errors (*I*/σ)^asymptotic^ (Diederichs, 2010 ▸, 2015 ▸) – herein abbreviated as ISa – and that for the random errors CC_1/2_ (Karplus & Diederichs, 2012 ▸, 2015 ▸; Diederichs & Karplus, 2013 ▸) in the highest-resolution shell that still has significant data (as revealed by a statistical test in CORRECT) can be used to make the decision. Those options that improve the statistics can then be used in combination.

After CORRECT, the data may be further analyzed using the ‘statistics’ tab (Fig. 4 ▸). The resulting XDS_ASCII.HKL may be converted to *e.g.* MTZ file format, using the ‘XDSCONV’ tab and *CCP4* programs. If other isomorphous data sets are available, the ‘XSCALE’ tab can be used to scale and merge them. Furthermore, XSCALE offers the possibility of zero-dose extrapolation which reduces the effect of radiation damage primarily for susceptible sites (Diederichs *et al.*, 2003 ▸).

Beyond starting, documenting and visualizing the progress of data processing, *XDSGUI* thus allows the user

(i) to inspect visually IDXREF’s indexed and non-indexed reflections in reciprocal space, which allows understanding and resolution of problems like multiple lattices

(ii) to identify beamline and crystal problems which are revealed by discontinuities in parameters plotted in the ‘INTEGRATE’ tab

(iii) to optimize the data processing parameters, by switching between ‘tools’ and ‘XDS.INP’ tabs, through a simple storage mechanism that allows the user to save, restore and compare the best results obtained so far

(iv) to address significant radiation damage using the ‘CORRECT’ and ‘statistics’ tabs, to decide on the frame range that should be accepted for scaling and merging

(v) to extend its functionality by running external programs or encoding user-defined operations in scripts that are executed upon the push of a button

### 3.2. Usage for *SHELX* data processing and phasing

**Figure 5:**
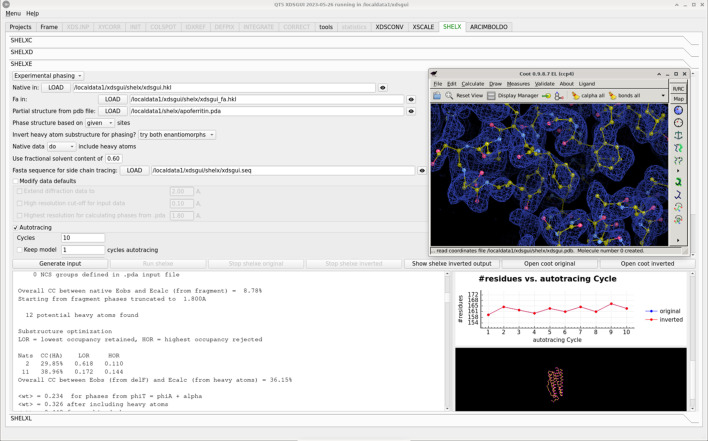
The ‘SHELX’ tab. The programs *SHELXC*, *SHELXD*, *SHELXE* and *SHELXL* are presented in different collapsible sections from top to bottom in the logical sequence of events. The label on the tab is green, indicating the run is in progress, in this case the solution of apoferritin 2g4h (Mueller-Dieckmann *et al.*, 2007 ▸). After data preparation with *SHELXC* and location of four Cd sites with *SHELXD*, phasing, density modification and model building are performed by *SHELXE*. The interface allows the user to modify the values inherited from the preceding programs or defaults or to introduce new parameters. In this case a partial structure is introduced as a PDB-format file with the extension .pda to be combined with the experimental phases. The output in the bottom left-hand window shows how *SHELXE* transforms the substructure to the consistent hand and origin fixed by the partial structure, which accounts for the results from the original and inverted substructures being identical, as seen in the right-hand plot. A Cα trace can be displayed as tracing progresses, or *Coot* can be called through the corresponding buttons to show the final map and model. A sequence was provided and side chains have been docked and traced.

The *SHELX* interface is presented within a single tab (Fig. 5 ▸), which is horizontally subdivided into expandable sections for *SHELC*, *SHELXD* (Schneider & Sheldrick, 2002 ▸), *SHELXE* (Sheldrick, 2002 ▸) and *SHELXL* (Sheldrick, 2015 ▸). The first three allow experimental phasing (Usón & Sheldrick, 2018 ▸), *e.g.* from an XDS_ASCII.HKL file produced previously in the same *XDSGUI* run, similar to *HKL2MAP* (Pape & Schneider, 2004 ▸), but offer the latest options of the programs, for example side-chain tracing (Usón & Sheldrick, 2022 ▸). *SHELXL* (Sheldrick, 2015 ▸) is a crystallographic refinement program, widely used in chemical and materials crystallography and also used in macromolecular crystallography for high-resolution structures. It has some unique features, such as calculation of standard uncertainties, flexible treatment of disorder and occupancy, and refinement of merohedral and non-merohedral twinning (Herbst-Irmer & Sheldrick, 1998 ▸; Sevvana *et al.*, 2019 ▸).

The ‘SHELXC’ section is the entry point for diffraction data. Selecting the type of experiment among SAD, SIR/RIP, SIRAS and MAD generates the necessary fields to load data sets, which will typically be in XDS_ASCII.HKL format but may include other general ASCII formats, like the *SHELX* .hkl format. The content of the files may be accessed through the GUI for viewing. Buttons labeled ‘Generate input’ and ‘run SHELXC’ configure *SHELXC* to prepare the files with anomalous differences or structure factor moduli estimated for the substructure. The tabular statistics output in *SHELXC* are displayed in plots, which should help in estimating the resolution limit to which the signal is significant and consistent.

The next section is labeled ‘SHELXD’ and will inherit the information from the files produced in the *SHELXC* run. The resolution cutoff should be limited to the shell where differences are still significant, as normalizing data upweights the high resolution. The number of sites may be uncertain initially, but after generating the input and on running *SHELXD* the value can be optimized by attending to the drop in figures of merit from one site to the next and in the lines of the minimum Patterson superposition function, displayed in the crossword table. A bimodal distribution in the correlation coefficient calculated for all data suggests a correct solution.

With a promising substructure, *SHELXE*, available in the next section, can derive, modify and extend phases to solve the structure. Both the determined substructure and its enantiomorphic configuration need to be tested, as direct methods cannot differentiate between them. Discrimination between the two runs is a positive sign. If a partial structure is known, it can be input in Protein Data Bank (PDB; Berman *et al.*, 2000 ▸) format and combined into the experimental phasing (MRSAD) (Panjikar *et al.*, 2009 ▸). *SHELXE* will refer the substructure to the same hand and origin, accepting the space group of the fragment. Model building options may be specified. Polyalanine tracing will be iterated, interspersed with density modification, and, if a sequence is provided, unspecific side-chain tracing will be used to improve phases, but side chains will only be docked and output once the correlation coefficient between observed and calculated normalized intensities has reached 30%. The *FASTA* format file should contain one copy of the sequence for each monomer expected in the structure.

The solution may be refined in the fourth section, labeled ‘SHELXL’. Generation of the *SHELX* format input file is mediated by the program *PDB2INS* (Lübben & Sheldrick, 2019 ▸).

### 3.3. Usage for *ARCIMBOLDO* data phasing

**Figure 6:**
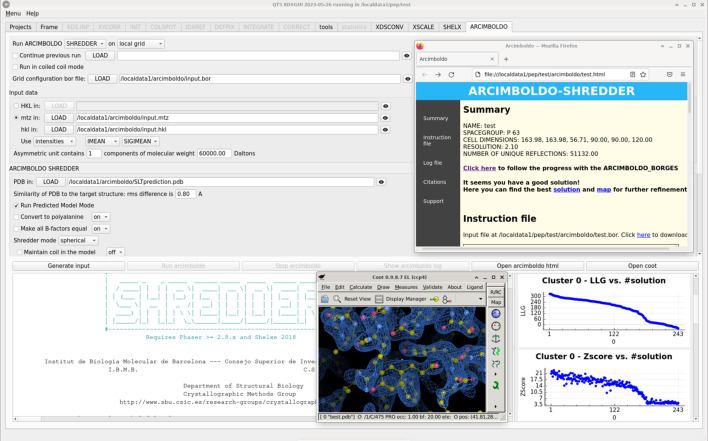
The ‘ARCIMBOLDO’ tab. The solution of the lytic transglycosylase Slt (Lee *et al.*, 2018 ▸) with a predicted model is displayed. The interface allows the user to select the *ARCIMBOLDO* mode, in this case *SHREDDER*, and the hardware to be used. The displayed calculations are distributed through a local grid for which the program has been given a configuration file. On the right-hand side, plots displaying figures of merit characterizing the results are displayed. The HTML *ARCIMBOLDO* output has been opened and the resulting solution is displayed in *Coot* by clicking the available buttons.

The ‘ARCIMBOLDO’ tab (Fig. 6 ▸) presents the general choices of which mode to use: *ARCIMBOLDO_LITE* (Sammito *et al.*, 2015 ▸), typically using one or more polyalanine helices as search models, *ARCIMBOLDO_BORGES* (Sammito *et al.*, 2013 ▸), using libraries representing variations of a local fold, or *ARCIMBOLDO_SHREDDER*, to identify, improve and use similar fragments to the unknown structure in a remote homolog or predicted model (Sammito *et al.*, 2014 ▸; Millán *et al.*, 2018 ▸). The more computationally intensive *ALIXE* (Millán *et al.*, 2020 ▸), *SHELXE* and *Phaser* jobs launched by *ARCIMBOLDO* may be run locally in multiple threads or distributed through a remote or local grid if one is configured, and the hardware to be used can be selected from the choices detected. The coiled coil mode can be activated for every mode to address the difficulties in discriminating correct from incorrect solutions that data modulation poses on the solution of coiled coils (Caballero *et al.*, 2018 ▸). Also, an interrupted run can be continued from the last checkpoint completed, which may be advantageous for a long calculation.

Once a mode is selected, the form will request the mandatory input and offer selection fields to customize parameters relevant to that mode. As for the other programs, input should be generated and the run launched. It is possible to terminate a run and display the terminal output, which in the case of *ARCIMBOLDO* is meant for troubleshooting. Results and access to map and PDB files for the best solution can be found in the HTML output common to all *ARCIMBOLDO* programs, rightmost of the buttons controlling job operations.

Accurate predicted models (Jumper *et al.*, 2021 ▸) provide a convenient entry to molecular replacement phasing (McCoy *et al.*, 2022 ▸). The predicted models mode in *ARCIMBOLDO_SHREDDER* (Medina *et al.*, 2022 ▸) may be selected from the interface. It has been optimized to solve the structure with predicted models and systematically eliminate model bias. If molecular replacement with *Phaser* is straightforward or a molecular replacement solution is input, phasing with fragments will be skipped and model bias elimination is entered directly.

## 4. Summary and outlook

During the past ten years, *XDSGUI* has been presented many times at crystallographic workshops in Europe, the Americas and Asia, and has been available for download through the XDSwiki. It has proved easy to use by novices in data processing and phasing, and was enthusiastically received by experienced crystallographers.

The *XDSGUI* interface is frequently acknowledged as being used for data processing and phasing. The underlying Qt framework is feature rich, performant, and well supported on Linux and macOS architectures.

We plan to maintain *XDSGUI* for the foreseeable future and to implement more functionality, such as interfacing the calculation of *R*_complete_ in *SHELXL* (Luebben & Gruene, 2015 ▸).

## References

[bb1] Agirre, J., Atanasova, M., Bagdonas, H., Ballard, C. B., Baslé, A., Beilsten-Edmands, J., Borges, R. J., Brown, D. G., Burgos-Mármol, J. J., Berrisford, J. M., Bond, P. S., Caballero, I., Catapano, L., Chojnowski, G., Cook, A. G., Cowtan, K. D., Croll, T. I., Debreczeni, J. É., Devenish, N. E., Dodson, E. J., Drevon, T. R., Emsley, P., Evans, G., Evans, P. R., Fando, M., Foadi, J., Fuentes-Montero, L., Garman, E. F., Gerstel, M., Gildea, R. J., Hatti, K., Hekkelman, M. L., Heuser, P., Hoh, S. W., Hough, M. A., Jenkins, H. T., Jiménez, E., Joosten, R. P., Keegan, R. M., Keep, N., Krissinel, E. B., Kolenko, P., Kovalevskiy, O., Lamzin, V. S., Lawson, D. M., Lebedev, A. A., Leslie, A. G. W., Lohkamp, B., Long, F., Malý, M., McCoy, A. J., McNicholas, S. J., Medina, A., Millán, C., Murray, J. W., Murshudov, G. N., Nicholls, R. A., Noble, M. E. M., Oeffner, R., Pannu, N. S., Parkhurst, J. M., Pearce, N., Pereira, J., Perrakis, A., Powell, H. R., Read, R. J., Rigden, D. J., Rochira, W., Sammito, M., Sánchez Rodríguez, F., Sheldrick, G. M., Shelley, K. L., Simkovic, F., Simpkin, A. J., Skubak, P., Sobolev, E., Steiner, R. A., Stevenson, K., Tews, I., Thomas, J. M. H., Thorn, A., Valls, J. T., Uski, V., Usón, I., Vagin, A., Velankar, S., Vollmar, M., Walden, H., Waterman, D., Wilson, K. S., Winn, M. D., Winter, G., Wojdyr, M. & Yamashita, K. (2023). *Acta Cryst.* D**79**, 449–461.

[bb2] Assmann, G., Brehm, W. & Diederichs, K. (2016). *J. Appl. Cryst.* **49**, 1021–1028. 10.1107/S1600576716005471 PMC4886987 27275144

[bb50] Berman, H. M., Westbrook, J., Feng, Z., Gilliland, G., Bhat, T. N., Weissig, H., Shindyalov, I. N. & Bourne, P. E. (2000). *Nucleic Acids Res.* **28**, 235–242. 10.1093/nar/28.1.235 PMC102472 10592235

[bb3] Caballero, I., Sammito, M., Millán, C., Lebedev, A., Soler, N. & Usón, I. (2018). *Acta Cryst.* D**74**, 194–204. 10.1107/S2059798317017582 PMC5947760 29533227

[bb4] Diederichs, K. (2006). *Acta Cryst.* D**62**, 96–101. 10.1107/S0907444905031537 16369098

[bb5] Diederichs, K. (2010). *Acta Cryst.* D**66**, 733–740. 10.1107/S0907444910014836 20516626

[bb6] Diederichs, K. (2015). *Nucleic Acid Crystallography: Methods and Protocols*, edited by E. Ennifer, Methods in Molecular Biology, Vol. 1320, pp. 147–173. New York: Springer.

[bb7] Diederichs, K. & Karplus, P. A. (2013). *Acta Cryst.* D**69**, 1215–1222. 10.1107/S0907444913001121 PMC3689524 23793147

[bb8] Diederichs, K., McSweeney, S. & Ravelli, R. B. G. (2003). *Acta Cryst.* D**59**, 903–909. 10.1107/s0907444903006516 12777808

[bb9] Emsley, P., Lohkamp, B., Scott, W. G. & Cowtan, K. (2010). *Acta Cryst.* D**66**, 486–501. 10.1107/S0907444910007493 PMC2852313 20383002

[bb10] Evans, P. R. (2011). *Acta Cryst.* D**67**, 282–292. 10.1107/S090744491003982X PMC3069743 21460446

[bb11] Herbst-Irmer, R. & Sheldrick, G. M. (1998). *Acta Cryst.* B**54**, 443–449.

[bb12] Jumper, J., Evans, R., Pritzel, A., Green, T., Figurnov, M., Ronneberger, O., Tunyasuvunakool, K., Bates, R., Žídek, A., Potapenko, A., Bridgland, A., Meyer, C., Kohl, S. A. A., Ballard, A. J., Cowie, A., Romera-Paredes, B., Nikolov, S., Jain, R., Adler, J., Back, T., Petersen, S., Reiman, D., Clancy, E., Zielinski, M., Steinegger, M., Pacholska, M., Berghammer, T., Bodenstein, S., Silver, D., Vinyals, O., Senior, A. W., Kavukcuoglu, K., Kohli, P. & Hassabis, D. (2021). *Nature*, **596**, 583–589.

[bb13] Kabsch, W. (2010*a*). *Acta Cryst.* D**66**, 125–132. 10.1107/S0907444909047337 PMC2815665 20124692

[bb14] Kabsch, W. (2010*b*). *Acta Cryst.* D**66**, 133–144. 10.1107/S0907444909047374 PMC2815666 20124693

[bb15] Karplus, P. A. & Diederichs, K. (2012). *Science*, **336**, 1030–1033. 10.1126/science.1218231 PMC3457925 22628654

[bb16] Karplus, P. A. & Diederichs, K. (2015). *Curr. Opin. Struct. Biol.* **34**, 60–68. 10.1016/j.sbi.2015.07.003 PMC4684713 26209821

[bb17] Kolenko, P., Stránský, J., Koval’, T., Malý, M. & Dohnálek, J. (2021). *J. Appl. Cryst.* **54**, 996–1005.

[bb18] Krissinel, E., Lebedev, A. A., Uski, V., Ballard, C. B., Keegan, R. M., Kovalevskiy, O., Nicholls, R. A., Pannu, N. S., Skubák, P., Berrisford, J., Fando, M., Lohkamp, B., Wojdyr, M., Simpkin, A. J., Thomas, J. M. H., Oliver, C., Vonrhein, C., Chojnowski, G., Basle, A., Purkiss, A., Isupov, M. N., McNicholas, S., Lowe, E., Triviño, J., Cowtan, K., Agirre, J., Rigden, D. J., Uson, I., Lamzin, V., Tews, I., Bricogne, G., Leslie, A. G. W. & Brown, D. G. (2022). *Acta Cryst.* D**78**, 1079–1089.

[bb19] Krissinel, E., Uski, V., Lebedev, A., Winn, M. & Ballard, C. (2018). *Acta Cryst.* D**74**, 143–151. 10.1107/S2059798317014565 PMC5947778 29533240

[bb20] Lee, M., Batuecas, M. T., Tomoshige, S., Domínguez-Gil, T., Mahasenan, K. V., Dik, D. A., Hesek, D., Millán, C., Usón, I., Lastochkin, E., Hermoso, J. A. & Mobashery, S. (2018). *Proc. Natl Acad. Sci. USA*, **115**, 4393–4398. 10.1073/pnas.1801298115 PMC5924928 29632171

[bb21] Liebschner, D., Afonine, P. V., Baker, M. L., Bunkóczi, G., Chen, V. B., Croll, T. I., Hintze, B., Hung, L.-W., Jain, S., McCoy, A. J., Moriarty, N. W., Oeffner, R. D., Poon, B. K., Prisant, M. G., Read, R. J., Richardson, J. S., Richardson, D. C., Sammito, M. D., Sobolev, O. V., Stockwell, D. H., Terwilliger, T. C., Urzhumtsev, A. G., Videau, L. L., Williams, C. J. & Adams, P. D. (2019). *Acta Cryst.* D**75**, 861–877.

[bb22] Lübben, A. V. & Sheldrick, G. M. (2019). *J. Appl. Cryst.* **52**, 669–673. 10.1107/S1600576719005478 PMC6557183 31236096

[bb23] Luebben, J. & Gruene, T. (2015). *Proc. Natl Acad. Sci. USA*, **112**, 8999–9003. 10.1073/pnas.1502136112 PMC4517205 26150515

[bb24] McCoy, A. J., Grosse-Kunstleve, R. W., Adams, P. D., Winn, M. D., Storoni, L. C. & Read, R. J. (2007). *J. Appl. Cryst.* **40**, 658–674. 10.1107/S0021889807021206 PMC2483472 19461840

[bb25] McCoy, A. J., Sammito, M. D. & Read, R. J. (2022). *Acta Cryst.* D**78**, 1–13. 10.1107/S2059798321012122 PMC8725160 34981757

[bb26] Medina, A., Jiménez, E., Caballero, I., Castellví, A., Triviño Valls, J., Alcorlo, M., Molina, R., Hermoso, J. A., Sammito, M. D., Borges, R. & Usón, I. (2022). *Acta Cryst.* D**78**, 1283–1293. 10.1107/S2059798322009706 PMC9629495 36322413

[bb27] Millán, C., Jiménez, E., Schuster, A., Diederichs, K. & Usón, I. (2020). *Acta Cryst.* D**76**, 209–220. 10.1107/S205979832000056X PMC7057212 32133986

[bb28] Millán, C., Sammito, M. & Usón, I. (2015). *IUCrJ*, **2**, 95–105. 10.1107/S2052252514024117 PMC4285884 25610631

[bb29] Millán, C., Sammito, M. D., McCoy, A. J., Nascimento, A. F. Z., Petrillo, G., Oeffner, R. D., Domínguez-Gil, T., Hermoso, J. A., Read, R. J. & Usón, I. (2018). *Acta Cryst.* D**74**, 290–304. 10.1107/S2059798318001365 PMC5892878 29652256

[bb30] Mueller-Dieckmann, C., Panjikar, S., Schmidt, A., Mueller, S., Kuper, J., Geerlof, A., Wilmanns, M., Singh, R. K., Tucker, P. A. & Weiss, M. S. (2007). *Acta Cryst.* D**63**, 366–380. 10.1107/S0907444906055624 17327674

[bb31] Oeffner, R. D., Afonine, P. V., Millán, C., Sammito, M., Usón, I., Read, R. J. & McCoy, A. J. (2018). *Acta Cryst.* D**74**, 245–255. 10.1107/S2059798318004357 PMC5892874 29652252

[bb32] Panjikar, S., Parthasarathy, V., Lamzin, V. S., Weiss, M. S. & Tucker, P. A. (2009). *Acta Cryst.* D**65**, 1089–1097. 10.1107/S0907444909029643 PMC2756167 19770506

[bb33] Pape, T. & Schneider, T. R. (2004). *J. Appl. Cryst.* **37**, 843–844.

[bb34] Potterton, E., Briggs, P., Turkenburg, M. & Dodson, E. (2003). *Acta Cryst.* D**59**, 1131–1137. 10.1107/s0907444903008126 12832755

[bb35] Potterton, L., Agirre, J., Ballard, C., Cowtan, K., Dodson, E., Evans, P. R., Jenkins, H. T., Keegan, R., Krissinel, E., Stevenson, K., Lebedev, A., McNicholas, S. J., Nicholls, R. A., Noble, M., Pannu, N. S., Roth, C., Sheldrick, G., Skubak, P., Turkenburg, J., Uski, V., von Delft, F., Waterman, D., Wilson, K., Winn, M. & Wojdyr, M. (2018). *Acta Cryst.* D**74**, 68–84. 10.1107/S2059798317016035 PMC5947771 29533233

[bb36] Rodríguez, D. D., Grosse, C., Himmel, S., González, C., de Ilarduya, I. M., Becker, S., Sheldrick, G. M. & Usón, I. (2009). *Nat. Methods*, **6**, 651–653. 10.1038/nmeth.1365 19684596

[bb37] Sammito, M., Meindl, K., de Ilarduya, I. M., Millán, C., Artola-Recolons, C., Hermoso, J. A. & Usón, I. (2014). *FEBS J.* **281**, 4029–4045. 10.1111/febs.12897 24976038

[bb38] Sammito, M., Millán, C., Frieske, D., Rodríguez-Freire, E., Borges, R. J. & Usón, I. (2015). *Acta Cryst.* D**71**, 1921–1930. 10.1107/S1399004715010846 26327382

[bb39] Sammito, M., Millán, C., Rodríguez, D. D., de Ilarduya, I. M., Meindl, K., De Marino, I., Petrillo, G., Buey, R. M., de Pereda, J. M., Zeth, K., Sheldrick, G. M. & Usón, I. (2013). *Nat. Methods*, **10**, 1099–1101. 10.1038/nmeth.2644 24037245

[bb40] Schneider, T. R. & Sheldrick, G. M. (2002). *Acta Cryst.* D**58**, 1772–1779. 10.1107/s0907444902011678 12351820

[bb41] Sevvana, M., Ruf, M., Usón, I., Sheldrick, G. M. & Herbst-Irmer, R. (2019). *Acta Cryst.* D**75**, 1040–1050. 10.1107/S2059798319010179 PMC6889912 31793898

[bb42] Sheldrick, G. M. (2002). *Z. Kristallogr.* **217**, 644–650.

[bb43] Sheldrick, G. M. (2008). *Acta Cryst.* A**64**, 112–122. 10.1107/S0108767307043930 18156677

[bb44] Sheldrick, G. M. (2015). *Acta Cryst.* C**71**, 3–8.

[bb45] Sparta, K. M., Krug, M., Heinemann, U., Mueller, U. & Weiss, M. S. (2016). *J. Appl. Cryst.* **49**, 1085–1092.

[bb46] Usón, I. & Sheldrick, G. M. (2018). *Acta Cryst.* D**74**, 106–116. 10.1107/S2059798317015121 PMC5947774 29533236

[bb47] Usón, I. & Sheldrick, G. M. (2022). *bioRxiv*:2022.04.28.489939.

[bb48] Winn, M. D., Ballard, C. C., Cowtan, K. D., Dodson, E. J., Emsley, P., Evans, P. R., Keegan, R. M., Krissinel, E. B., Leslie, A. G. W., McCoy, A., McNicholas, S. J., Murshudov, G. N., Pannu, N. S., Potterton, E. A., Powell, H. R., Read, R. J., Vagin, A. & Wilson, K. S. (2011). *Acta Cryst.* D**67**, 235–242. 10.1107/S0907444910045749 PMC3069738 21460441

